# Precision Oncology Beyond Genomics: The Future Is Here—It Is Just Not Evenly Distributed

**DOI:** 10.3390/cells10040928

**Published:** 2021-04-17

**Authors:** Ulrike Pfohl, Alina Pflaume, Manuela Regenbrecht, Sabine Finkler, Quirin Graf Adelmann, Christoph Reinhard, Christian R. A. Regenbrecht, Lena Wedeken

**Affiliations:** 1 CELLphenomics GmbH, Robert-Rössle-Str. 10, 13125 Berlin, Germany; ulrike.pfohl@cellphenomics.com (U.P.); alina.pflaume@cellphenomics.com (A.P.); christoph.reinhard@cellphenomics.com (C.R.); quirin.adelmann@asc-oncology.com (Q.G.A.); christian.regenbrecht@asc-oncology.com (C.R.A.R.); 2 ASC Oncology GmbH, Robert-Rössle-Str. 10, 13125 Berlin, Germany; sabine.finkler@asc-oncology.com; 3 Institut für Molekulare Biowissenschaften, Goethe Universität Frankfurt am Main, Theodor-W.-Adorno-Platz 1, 60323 Frankfurt am Main, Germany; 4 Helios Klinikum Berlin-Buch, Schwanebecker Chaussee 50, 13125 Berlin, Germany; manuela.regenbrecht@helios-gesundheit.de; 5 Institut für Pathologie, Universitätsklinikum Göttingen, Robert-Koch-Straße 40, 37075 Göttingen, Germany

**Keywords:** intra-tumor heterogeneity, multi-omics technology, cancer models, patient-derived organoids, personalized oncology

## Abstract

Cancer is a multifactorial disease with increasing incidence. There are more than 100 different cancer types, defined by location, cell of origin, and genomic alterations that influence oncogenesis and therapeutic response. This heterogeneity between tumors of different patients and also the heterogeneity within the same patient’s tumor pose an enormous challenge to cancer treatment. In this review, we explore tumor heterogeneity on the longitudinal and the latitudinal axis, reviewing current and future approaches to study this heterogeneity and their potential to support oncologists in tailoring a patient’s treatment regimen. We highlight how the ideal of precision oncology is reaching far beyond the knowledge of genetic variants to inform clinical practice and discuss the technologies and strategies already available to improve our understanding and management of heterogeneity in cancer treatment. We will focus on integrating multi-omics technologies with suitable in vitro models and their proficiency in mimicking endogenous tumor heterogeneity.

## 1. Introduction

Despite all advancements in research and clinical practice, cancer remains a life-threatening disease with increasing incidence. Based on a prognosis by the WHO in 2018, cancer incidence is expected to double to about 37 million new cancer cases by 2040 [1].

Effective disease management is critical to cancer treatment. Current cancer treatments have made enormous progress from the first cytotoxic agents aiming at replicating cells via targeted therapies selectively aiming at genomic aberrant pathways like cetuximab for the treatment of advanced colorectal cancer to immuno-targeted drugs like ipilimumab for the treatment of malignant melanoma [2,3].

Despite the progress in medical oncology, most cancers are still treated by surgical resection of the tumors if accessible [4]. Pivotal to subsequent adjuvant or neo-adjuvant chemotherapy is the application of strict guideline protocols (S3 guidelines in Europe). These protocols are based on studies of large patient cohorts with similar cancers. This approach has led to a significant increase in progression-free (PFS) and overall survival (OAS) of patients, yet a majority of patients does not fully benefit from the administered treatment regimen [5]. The reason for therapeutic regimens falling short in those patients is the fact that every cancer comes with an individual, virtually unique genetic landscape [6].

Until it will become possible to faithfully predict the individual outcome of a specific treatment, oncologists and patients alike experience painful uncertainty regarding therapy success at the start and course of treatment. Even worse, both parties know that even when the tumor does not regress, the dire side effects of treatment will still impact the patient’s quality of life.

It is the aim of precision oncology to overcome this dilemma. Understanding the interplay between the unique characteristics of a patient’s tumor and the medical treatment and how it can be tailored to the individual properties of the tumor is a major focus in translational cancer research. Today, cancer precision medicine mostly aims at matching specific tumor mutations with drugs targeting aberrant oncogenic pathways to provide individualized treatment options relying on small organic compounds and/or monoclonal antibodies [7]. While most cancers harbor multiple oncogenic mutations, preclinical and clinical data support the idea that many cancers are sensitive to inhibition of single oncogenes, a concept referred to as ‘oncogene addiction’ [8].

This mutation-driven approach to cancer precision medicine is also applied to pre-therapeutic cohort stratification, which has subsequently led to the concept of conditional approvals, i.e., certain, targeted cancer therapeutics are only approved for patients with a defined set of specific mutations.

The precedent of a targeted cancer therapeutic with conditional approval is the anti-epidermal growth factor receptor (EGFR) antibody cetuximab (Erbitux^®^). In colorectal cancer (CRC), examination of molecular alterations indicated that mutations in *KRAS*, which is downstream from EGFR in the RAS-MEK-ERK signaling pathway, interfered with this therapy [9,10]. Cetuximab is therefore only relevant for *RAS* wildtype tumors. Following the success of cetuximab, other small molecules, biosimilar and monoclonal antibodies were investigated and successfully applied in the clinic. Today, our arsenal of targeted drugs in oncology comprises 84 approved agents [11].

Yet, cancer is far from being conquered, reflected by the fact that cancer is the second leading cause of death worldwide accounting for an estimated 9.6 million deaths in 2018 [12]. In addition, it becomes more and more evident that the genetic approach to cancer precision medicine alone is not sufficient to predict individual treatment response. This is mainly due to intra-tumor heterogeneity, which is currently not sufficiently taken into consideration.

## 2. Intra-Tumor Heterogeneity—The Challenge of Treating “Many Cancers in One”

The biology of cancer is complex and not yet fully understood. During malignant transformation tumors may acquire increasingly aggressive features and over time increase their metastatic potential and propensity to gain treatment resistance [13,14]. These hallmark features develop by clonal evolution which is fueled by the complex interplay of cancer cells and their microenvironment [15]. This unique composition of any given tumor is one of the biggest clinical challenges in modern oncology [16,17].

At the time of diagnosis, the tumor usually has passed millions to billions of aberrant cell divisions that frequently lead to genetic instability and genomic and epigenetic heterogeneity [18]. The path of malignant transformation from benign hyperplasia (non-cancerous) to malignant (cancerous) may follow different routes of clonal evolution [19]. The later the tumor has been diagnosed the higher degree of intra-tumoral diversity is expected [13]. Heterogeneity happens latitudinally between tumors from different patients (inter-patient heterogeneity) and longitudinally in the tumor (and metastases) of the same patient (intra-patient heterogeneity) [20]. A well-described example for heterogeneity is the hepato-cellular carcinoma (HCC) with a high degree of longitudinal heterogeneity between patients as well as latitudinal heterogeneity within the same tumor of an individual patient [21].

An emerging field is the immunogenic heterogeneity, which is common in liver tumors but not limited to this tumor entity [22,23]. Tumors from different patients show a different degree of immune cell infiltration and immune cell composition [23]. Immunologically “hot” tumors present high levels of T cell infiltration [24]. In consequence, these tumors are more susceptible to immune-checkpoint inhibitor treatment as compared to immunologically “cold” tumors [24]. This immunogenic heterogeneity impacts treatment outcome. There are multiple studies ongoing which aim to define a consensus classification for molecular (immune) subclasses [22,25].

Intra-patient heterogeneity or intra-tumor heterogeneity (ITH) has been proven for solid tumors and usually refers to genetic changes within cell subpopulations that form the tumor mass after multiple cell divisions and proliferation during tumor growth [26,27,28,29]. Thus, solid tumors can be described as heterogeneous neoplasms comprised of different types of cells. A representative solid tumor is composed of malignant cells, communized with mesenchymal cells, endovascular cells, and immune cells creating the tumor microenvironment [30].

ITH is the result of rather complex events and context, related to different causes and different outcome patterns. There are different types of ITH: morpho-histological [31,32], clonal [15] and nonclonal ITH [33,34,35].

Morpho-histological ITH is represented by different morphological structures, is related to different levels of differentiation and/or correlates with different molecular alterations, which was shown in lung adenocarcinomas where the mutant allele frequencies were higher in solid areas of the same tumor [36]. In breast cancer for example, morphological ITH was found to be associated with epithelial–mesenchymal transition (EMT) and stemness of cancer cells [32].

Nonclonal ITH derives from microenvironment interaction which makes the clinical approach challenging [31]. The tumor microenvironment is in constant chemical and physical interaction with the actual tumor. These interactions vary between the different areas of the primary tumor. Further, these interactions may not only fuel heterogeneity amongst the tumor cells, but also the stroma can become increasingly heterogeneous [33]. Besides paracrine signaling of cancer and stroma cells, the interaction with the different types of collagen within the stroma may impact therapy success [34,35].

Clonal ITH derives from genomic instability [37]. Different individual tumors of the same entity may undergo very different paths of clonal evolution, which was shown in liver cancer [38]. ITH is the basis for the selection of the fittest clones, a key step in clonal evolution [15].

Following the development of new analytic technologies, recent data support the clonal evolution model as the main theoretical basis of tumor heterogeneity [19,39].

A recent study from Yang et al. in patients with glioblastoma (GBM) provides a good understanding of intra-tumor heterogeneity and its impact on disease progression and recurrence. Regions from within the tumor were sequenced as well as blood-derived circulating tumor DNA from patients with primary and recurrent GBM [40]. They observed high intra-tumor heterogeneity at the levels of both somatic gene mutations and chromosomal copy numbers. In total, they analyzed over 1000 genes that are involved in tumorigenesis and cancer progression [40].

Another example revealing substantial ITH in a solid tumor is a recent study by Schumacher et al. using multi-region sampling of colorectal cancer. They generated five patient-derived three-dimensional (PD3D) “sibling” in vitro cultures from a single colorectal tumor and modelled consequences of ITH on tumor cell growth and drug response [41]. The heterogeneity of tumor tissues and PD3D sibling cultures was further evaluated at DNA and mRNA levels. Using cancer panel-sequencing, common mutations were detected (KRAS^G12D^, PIK3CAH^1047R^, and TP53^C242F^) in the tumor tissue-of-origin and the separate sibling cultures [41]. They found an additional homozygous SMAD4^R361H^ mutation in two out of the five regions which might be responsible for different drug responses of the different cultures. Not only did drug responses of sibling cultures show up to a 30-fold difference, but also substantial heterogeneity in mRNA expression of target genes of ERK/MAPK, PI3K and mTOR signaling pathways was observed [41]. Such investigations are crucial for the acceptance of testing patient-derived models as they aim to understand the heterogeneity of tumors in the clinical context. This will offer easy access to molecular “toolboxes” in translational oncology to learn how to overcome treatment resistance.

The impact of ITH on drug resistance and targeted therapy strategies has been demonstrated using multiple-site profiling in solid tumors and is regarded as a paradigm shift in cancer care [42]. In contrast, only a limited number of studies addressed ITH in non-solid tumors [43,44,45,46]. Wogsland et al. studied intra- and inter-tumor heterogeneity of follicular lymphoma (FL), a B-cell malignancy, by using mass cytometry to obtain deep profiling of cell subsets [43]. This study allowed the identification of biologically relevant features including tumor heterogeneity and loss of non-malignant B-cell subsets. Additional proteins with a high variability among lymphoma cells have been identified in the same tumor [43].

In a proof-of-concept study, Araf et al. performed a combination of whole-exome and targeted deep sequencing of spatially and temporally separated biopsies from patients with follicular lymphoma [44]. Their results suggest that the analyzed tumors consisted of multiple subclones. Different tumor subpopulations dynamically circulated in the plasma with increasing heterogeneity during malignant transformation. Yet, their data did not show an association between increasing genetic ITH and therapy success [44]. Nevertheless, these results warrant further studies about ITH in liquid tumors to understand its relevance in the clinic.

In summary, ITH has been identified as being causal for the often-observed failure of current tumor therapies, especially in solid tumors. In addition, ITH might limit the reproducibility in clinical cancer research and precise diagnostic evaluation of tumors [47]. Therefore, ITH is a major challenge of cancer treatment which needs to be considered in precision cancer medicine.

## 3. Integration of Intra-Tumor Heterogeneity into Multi-Layered Personalized Cancer Therapy

Regardless of the tumor evolution that is causing ITH, the existence of multiple distinctive cell populations in the same tumor has strong clinical implications [48]. The diagnosis of primary and metastatic tumors is typically based on a single biopsy representing only a snapshot of ongoing tumor evolution and may be compromised by ITH [49]. The studies described above show that a sole biopsy does not accurately capture the tumor’s genetic and phenotypic heterogeneity [28,46]. Therefore, multimodal strategies have to be taken into account to ensure patients receive effective and targeted therapy. In addition, it is necessary to develop new tools to study heterogeneity, and to identify new biomarkers of tumor heterogeneity. Further, besides focusing on clonal heterogeneity, nonclonal phenotypic heterogeneity should be taken into consideration, i.e., the fact that some cells respond to broad, environmental perturbations and drug treatments by conversion to many other cell states, including stem-like, resistant cell phenotypes [50]. Finally, suitable models are needed that also take the effect of the tumor microenvironment into account.

### 3.1. Current Approaches to Analyze ITH from Tumor Samples

Still, most efforts of molecular tumor boards predominantly focus on genomic sequence information to diagnose patients, to predict the individual risk of developing cancer, and to assess whether specific treatments are suitable (i.e., likely to be successful) [7]. The concept of ITH is only rarely covered in these boards.

Major advances in the concept of tumor heterogeneity have come with the implementation of high-throughput genomic sequencing technologies. These allow the profiling of chromosomal and point mutations of neoplasms, with genomic data being most commonly used for molecular cancer diagnostics for rational clinical decision making [32].

However, intra-tumoral heterogeneity results from much more complex interplay on different levels. Genetic, epigenetic, and protein modifications are determining the phenotype of a given tumor. Plasticity allows for adaption in response to environmental factors by modulation of cellular properties [51]. These complexities are very well put into conceptual context by Brock and Huang [52]. Recent studies showed that only about 10% of cancer patients benefit from genomic sequencing of their tumor [53]. This argues the use of a single technology for decision making but suggests a multi-omics approach to provide deeper integration of precision oncology into the clinic in order to pave the way for tailored therapy schedules for the benefit of patients and doctors alike.

#### 3.1.1. Genomic Approaches to ITH and Precision Oncology

Today, precision oncology is based on the assumption that tumor treatment is more effective when selecting a target-specific therapy that matches the genetic or epigenetic changes observed in a single tumor of a cancer patient [13].

Since the genomic revolution more than 30 years ago, array-based and sequencing approaches have been used extensively to identify and characterize the molecular events underlying cancer development and progression [54]. Today, many of these learnings are widely used for diagnostics. Large multinational consortia like “The Cancer Genome Atlas” (TCGA) initiative provide datasets of individual tumors and tissues from thousands of patients from a wide array of cancers providing insights into the complexity of cancer [55]. It was found that mutations within the same tumor differ enormously in their allelic frequencies [56]. Based on the grouping of allelic frequencies, an estimate of the genetically different “clones” of cancer cells can be made for each tumor [56].

The presence of multiple clones within the same tumor sparked an ongoing discussion about the need of multi-regional sampling to discover ITH in tumors. Surprisingly, Zhang et al. concluded that complete assessment of ITH complexity may not require sampling in multiple regions [57]. They showed for lung adenocarcinoma that a single biopsy may be sufficient to capture the majority of mutations if ultra-deep sequencing is performed [57]. This is in stark contrast to other studies like the one by Gerlinger et al. [58]. Multiregional sequencing on samples obtained from primary renal carcinomas and associated metastatic sites demonstrated substantial ITH, with several mutations in certain cancer genes being restricted to separated tumor domains [58]. These studies suggest that a single biopsy may not be suitable for the identification of all cancer gene mutations of a tumor, thus providing an incomplete view of potential targets for cancer therapy [13,58,59].

Surprisingly, several clinical trials driven by genomics have been resulting in positive response rates of only 5–10% [53]. Among those studies, the most successful study was the WINTHER trial (NCT01856296). During the six-year study, patients were stratified to different treatments based on DNA-sequencing from fresh biopsy-derived tissue (arm A; 236 gene panel) vs. RNA expression analysis (arm B; comparing tumor to normal). Based on the sequencing results, treatment recommendations were made by a multi-national “clinical management committee” with subsequent monitoring of the applied therapy by oncologists. Treatment scores were calculated for each patient based on an array of clinical parameters. Within the patient cohort included in the study, stable disease (≥6 months) and partial or complete response was 26.2% (arm A: 23.2%; arm B: 31.6%) compared to previous treatment regimen progression-free survival of 22.4% [60]. The most important predictor of successful treatment was actually fewer previous therapies, while genomic and transcriptomic profiling could only marginally improve treatment recommendations and patient outcome [61].

While genomic analysis is invaluable for our current understanding of cancer and the development of better therapies, sequencing alone is not sufficient for tailored therapies. These molecular snapshots cannot answer clinically relevant questions: does a specific alteration occur in all cells or just in a small part of the tumor? How many regions of a tumor should be sequenced to get a representative result? In addition, the presence or absence of a specific mutation is not always sufficient to predict the effect of a targeted therapy. This is well documented in colorectal cancer, where *RAS* mutations are used to estimate the potential success of anti-EGFR treatment [62]. While anti-EGFR competitors like cetuximab or panitumumab correspond with a longer overall survival of patients with *RAS* wild-type, not all *RAS* wild-type patients benefit from upstream RAS inhibition due to the tissue context [63,64,65,66,67,68]. Patients with right hemi colic cancers do not benefit from anti-EGFR therapy irrespective of RAS status [69]. Current guidelines take such spatial heterogeneity into account, and therefore suggest the use of cetuximab or panitumumab only for patients with *RAS* wild-type and left-sided colon cancer [70,71].

#### 3.1.2. Proteomic Approaches

During the last few decades, not only have sequencing technologies advanced significantly, but other technologies such as proteome and metabolome analyses have also become more suitable for a deeper characterization of tumor cells and their functional abnormalities [72,73].

Transcriptomics capture the quantity of the immediate product of a cell’s genome at a given timepoint. It is a snapshot of the genomic expression at a certain time, or the change thereof over time or under different conditions [74]. In contrast, proteomics dissect the cellular protein composition, the result of gene translation [75]. Initial large-scale studies of cellular proteomes showed a relatively low correlation between protein expression levels and corresponding mRNA expression levels [64].

As on the genomic level, protein function is mediated and altered by multiple mechanisms. Posttranslational modifications of proteins (e.g., phosphorylation) are often required for their activity or signaling. Folding and posttranslational processing of proteins is a prerequisite for the formation of multi-protein complexes, which in turn are necessary to act as molecular machines, performing almost all cellular functions. To add another layer of complexity, the cellular location of a specific protein often determines whether the protein is active or inactive, or if a protein complex is successfully formed. In cancer proteomics, the TCGA Consortium represents the first large-scale effort to profile the tumor proteome [76]. However, the analysis was performed using reverse phase protein arrays, and was therefore limited to the targeted analysis of a few hundred proteins.

Despite the complexity, proteomic approaches have led to the identification of specific biomarkers in ovarian cancer [65] and identification of molecular subgroups in breast cancer [66]. Proteomic data used in precision oncology has helped to correctly predict drug sensitivity and resistance [67,68]. Cancer proteomics can therefore be seen as complementing the traditional immunohistochemical classification of tumor types, such as the characterization of estrogen receptor expression in breast cancers [77,78].

#### 3.1.3. Metabolomic Approaches

Metabolites are the products of cellular processes, which in turn are driven by proteins, mostly enzymes. Therefore, changes in metabolites mirror changes in the activity of enzymes and proteins and may pose as ideal biomarkers [79]. Due to their accessibility, many metabolomic analyses are performed on plasma or serum samples from patients used in diagnosis without requiring an invasive intervention to obtain tumor tissue [80,81]. Yet, this analysis of individual levels of metabolites makes it difficult to determine universal levels and individual changes for a particular tumor entity. Currently, only few studies have been conducted, and validation is pending [82]. Among the few examples of a clinically relevant metabolomic approach is the use of metabolites for the identification of altered carbohydrates in acute myeloid leukemia [83], as well as unsaturated free fatty acids in colorectal cancer [84].

Given the limitation of current knowledge on metabolomics in cancer in general, we are still far away from understanding how it may contribute to a better understanding of ITH from measuring metabolomic changes in peripheral blood and how this is representative for the complexity of the tumor tissue.

Nevertheless, with the rise of genomic medicine and in-depth characterization of the individual tumor mutation landscape, a better understanding of tumor complexity and ITH suggest that the ‘precision medicine’ paradigm of cancer therapy requires multi-modal treatment to be personalized to the individual patient.

### 3.2. Suitable In Vitro Strategies for Modeling Intra-Tumor Heterogeneity

Utilizing cancer models may complement or even supplement the approaches described above. ITH, the cellular interactions and the tumor’s molecular alterations could be investigated in cancer models derived from patients’ samples to study the underlying mechanisms and/or learn how ITH may direct clinical decision making.

Cancer models are naturally existing or artificially induced systems that share characteristics with human cancers [85]. Experimental systems for the study of human cancer include genetically engineered mouse models (GEMMs) [86,87,88,89], two-dimensional (2D) cell lines [90], patient-derived organoids (PDO) [91,92,93,94,95], and patient-derived xenografts (PDX) [95,96] to study biochemical or genetic pathways and pathology of cancer. These in vivo and in vitro cancer models have been invaluable for our current understanding of cancer development and progression, as well as for therapy development. Further, these models are moving into focus regarding their potential use in cancer precision and/or personalized medicine [97,98].

Given the complexity and heterogeneity of cancer, a crucial question to be asked is whether these models are feasible to capture and investigate ITH.

GEMMs are created by inducing specific mutations in oncogenes and/or tumor suppressor genes and can be used to monitor tumorigenesis in vivo, but are limited by species differences in oncogenic pathogenesis, the shorter lifespan of mice, and often by the artificial simultaneous introduction of several oncogenic driver events [88,89].

Traditional 2D cell lines grow as monolayer, cultured on flat and rigid substrates [91]. They have the advantage that they have once been derived from a cancer patient and are easier to manipulate in the laboratory, but they cannot completely replicate the environment of the patient tumor. Even within the same cancer model, data between laboratories are often irreproducible [99,100]. Nevertheless, cancer 2D cell lines have been used not only for in vitro but also for in vivo experiments, for example to generate xenograft models by subcutaneous injection of cancer cell lines into immunodeficient mice [101].

Patient-derived xenografts (PDX) are generated by implantation of cancerous tissue from a patient’s tumor either under the skin (ectopic) or into the organ of tumor origin (orthotopic) and are most commonly used for preclinical drug development [102,103,104].

Physiologically, cells grow in three dimensions (3D) to form discrete tissue and organ structures [105,106]. PDO models, growing in 3D, have been shown to reliably recapitulate the architecture of the donor tissue and to preserve its genomic background, therefore providing a highly relevant physiological system [107]. With their optimal conditions for cellular proliferation, differentiation, and responsiveness to chemo- and targeted therapeutics, they recapitulate the functional tumor phenotype, including its ITH [41].

The impact of adding a third culture dimension on the cellular drug response has been shown by Koch et al. [108]. They compared the response of 2D colorectal cancer cell lines and 3D CRC cell line-derived spheroids to irradiation and chemotherapy [108]. 3D CRC cell line cultures were more resistant to irradiation and chemotherapy, such as 5-FU and cisplatin, than their 2D counterparts [108]. This must be taken into account when translating the results into clinical setting. Therefore, PDOs are increasingly used for studying tumor biology and the effects of targeted therapies [109].

Schütte et al. investigated a colorectal cancer biobank comprising PDX models and PDO models, which were treated with clinically relevant compounds [95]. They showed that PDOs recapitulate many of the genetic and transcriptomic features of the donor tumors whereas clonal discordances found at early passages were attributed to ITH [95]. Ben-David et al. showed that the genomic and transcriptomic heterogeneity of cell lines impairs our ability to evaluate new therapeutics [110]. Their results support efforts to systematically develop PDO models to reduce the reliance on poorly defined cell lines that were established before the next generation sequencing era [110]. Further, Vlachogiannis et al. showed that PDOs can recapitulate patient responses in the clinic and could therefore be implemented as models for personalized oncology [111].

**Figure 1:**
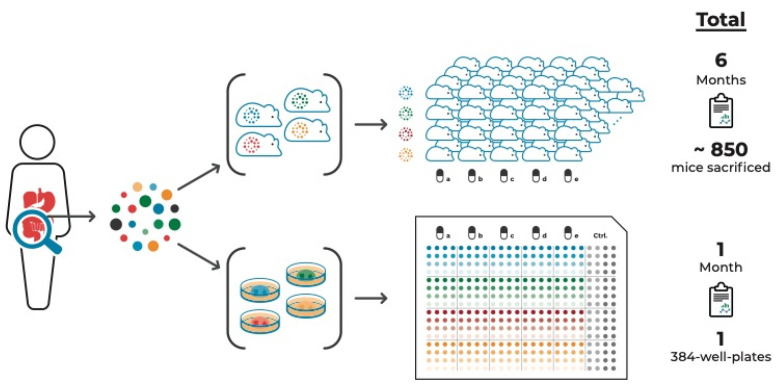
PDO vs. PDX cancer models to study a tumor’s drug response incorporating intra-tumor heterogeneity. Multi-regional sampling of a tumor is required to take ITH into account when assessing the tumor’s drug response. Compared to multi-regional PDX models, multi-regional PDO-based pre-therapeutic drug screenings are significantly more time- and cost-effective. These PDO-based screens, in addition to amplicon sequencing of multiple areas of tumor tissue and derived cell culture models, combined with targeted proteomic approaches, are both feasible and available within a timeframe that allows discussing guided treatment options.

An additional advantage of PDO cultures is that they can be used in high-throughput drug screens. Such screens can be composed of multiple samples from the same tumor, thereby taking ITH into account [41,94]. Figure 1 compares this approach with a PDX approach aiming to test the same number of tumor samples and compounds to illustrate the time- and cost-effectiveness of the organoid system.

Nevertheless, PDO are an in vitro culture system, therefore lacking, for example, liver or kidney clearance or liver pro-drug activation mechanism. Further, only the effect of the drug on the tumor (i.e., tumor-derived organoid) itself can be assessed, omitting potential toxicity on other organs or indirect effects asserted on, e.g., vasculature or hormone production in the pituitary gland. These aspects need to be taken into consideration for experimental design.

It is known that the microenvironment with tumor-surrounding and infiltrating cells, including fibroblasts and immune cells, have a major impact on drug response [112,113]. Another significant advantage of the organoid model system is the ability to study the interaction of cancer organoids with other specific cell types that can be introduced into a direct or indirect co-culture system. Indirect co-culture systems are based on the use of cell conditioned media. They are simple to apply and are therefore often used for in vitro experiments [114]. However, they are not suitable for investigating the effects of cell contacts between cancer cells and stroma [115]. Direct co-culture models are a closer representation of the in vivo scenario. Since the tumor microenvironment plays a critical role in tumorigenesis, 3D co-culture systems are used, including not only cancer cells but also stromal cells [115]. Cancer cells were localized in a defined area within a stromal cell matrix to study the cytotoxic effect of anticancer drugs on both tumor and normal cells in the same system [116].

Since antibodies against immune checkpoint proteins/receptors have shown clear clinical benefit for patients with advanced cancer, including melanoma, non-small cell lung cancer (NSCLC), and mismatch repair deficient (dMMR) colorectal cancer, organoid co-culture systems including immune cells are moving into the spotlight of current in vitro application [117,118,119,120,121,122,123,124,125,126]. Dijkstra et al. established a co-culture system of autologous tumor organoids and peripheral blood lymphocytes of patients to induce and analyze tumor-specific T cell responses for mismatch repair deficient colorectal cancer and non-small cell lung cancer in a personalized manner [118]. Klein et al. demonstrated the advantages of co-culture systems of GBM organoids and human immune cells, to investigate not only immune–tumor interactions, but also to explore current and novel immunotherapies, such as adoptive T cell transfer, immune checkpoint inhibitors, or oncolytic viruses [127].

Another promising technology for studying ITH is Organ-on-a-Chip (OoC), a culture model to mimic complex and dynamic in vivo microenvironments [128]. An OoC is a multi-channel 3D microfluidic biochip, which recapitulates the activity, mechanism, and pathophysiological reaction of single-organ and multi-organ systems [129]. It is a useful tool in controlling spatial arrangement of cell growth and fluids within micrometer-sized channels, which may be used to increase the physiological relevance of tumor models [130]. OoC technology is expected to offer effective solutions to investigate the effects of drugs, as well as the causes of diseases and personalized therapeutic treatments [131,132,133].

The development of a multi-organoid platform that consists of patient-specific tumor organoids is currently in process. It is intended to offer the opportunity to test the efficiency of drug therapies designed based on genetic profiling. Skardal et al. generated a circulatory system with multiple tissue organoid sites by using microfluidic chip devices and used them to visualize and track tumor progression and kinetics of metastasis formation to distant site in vitro [132,133]. In combination with the even more complex body-on-a-chip platform, these personalized on-a-chip systems will be improved even further [132,133].

This very promising technology offers great potential for in vivo tumor-like model systems to enable personalized drug screenings before treating patients and monitoring of organ systems in the OoC device for side effects at the same time. This new technology is still in development but has the potential to improve cancer treatment outcomes and patient care dramatically.

In summary, there are various suitable preclinical cancer models each with its own limitations. Despite this, these models are an attractive alternative or addition and have the potential to augment genetic and multi-omics approaches when considering ITH for precision cancer medicine.

## 4. Outlook

Currently, in the minds of many, precision oncology is a genomics-only approach. To efficiently assess ITH of tumors, it is essential to systematically integrate molecular patterns, protein expression, and morphology into the broader context of all available clinical and pathological information [134]. Another major aspect adding to the treatment conundrum are those cancer cells that survive treatment. These cells already underwent cytotoxic stress and were primed to resemble a stem-like cell state [135]. This pool of cells is commonly causing recurrence [135,136,137]. As these stages are not connected by a precise consecutive relationship, evidence suggests that even when preclinical and clinical responses concur, tumor heterogeneity remains a severe obstacle for routine translation of preclinical data into clinical practice [52].

The evaluation of the overall profile of gene expression, epigenetic alterations, and various regulatory elements is likely to be performed soon in clinical practice along with proteomic and metabolomic measurements [138,139]. The multi-omics knowledge bridges the gap between underlying molecular changes and cell behavior and facilitates a deeper understanding of disease development processes. As of today, a comprehensive multi-omics analysis of all cancer patients is unfeasible. The generation of multi-layered omics remains expensive and time consuming. The limited understanding of the biologic connections between the cells, their microenvironment, the tumor and the rest of the organism make such data extremely difficult to interpret and put into context. The availability of suitable tissue samples, high-quality biopsy material, and universal protocols to compare the results from different clinical sites and patients is crucial for a reliable analysis of the transcriptome, epigenome, proteome, and metabolome [140]. Steering away from the mere analysis of RNA and DNA towards the collection of living samples is essential, as analysis of nucleic acids requires fixation in buffers that inherently make the tissue sample unusable for protein profiling, metabolite detection, and ultimately viable patient-derived models [140].

Careful evaluation of current studies will show if we can generate the necessary comprehensive multi-omics data and convert it into meaningful and, thus, actionable information that allows to better understand and predict tumor formation, progression, development of resistance to treatment, and the risk of recurrence.

**Figure 2:**
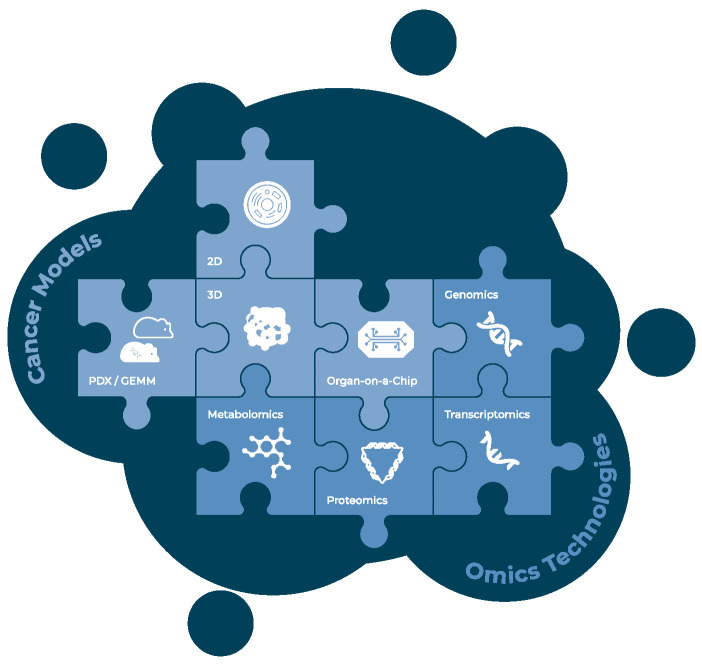
Cancer models and omics technologies to obtain a comprehensive picture of biological processes within a patient’s tumor. An appropriate combination of different omics technologies and relevant tumor models allows a more comprehensive analysis of intra-tumor heterogeneity, relevant to improve patient treatment. (PDX—patient-derived xenograft; GEMM—genetically engineered mouse models; 2D—two-dimensional cell culture; 3D—three-dimensional cell culture).

Beyond the generation of data and the potential use of sophisticated in vitro models, the even more challenging task is to identify and select the most meaningful omics data types to apply (limited primarily by time, cost and tissue availability) and in a second step to establish best-practice approaches to integrate the different datasets to obtain a comprehensive picture of the underlying biological processes (Figure 2). Very recent publications evaluating omics-based strategies for guiding the clinical management of difficult-to-treat tumors fuel hope to overcome the challenges of ITH [141]. In the long run, applying multiple omics-technologies may have the same significance for deciphering intra-tumor heterogeneity as the Rosetta stone had for learning how to read and interpret ancient hieroglyphs.

One possible approach is based on existing knowledge of the specific molecular pathways that are crucial to cancer development, progression, dissemination of metastases, and treatment response. Another approach would be an agnostic one, which aims to identify correlations across multiple data sets to determine altered molecules, analytes, or pathways. The benefit of this approach is its ability to discover novel molecules and pathways essential for the development and/or progression of disease.

In both cases, suitable cancer models are invaluable tools as they have the potential to overcome the issue of limited tissue availability and can be manipulated to address specific research questions, e.g., through manipulation of gene sequences and/or expression or comparison of untreated vs. treated cells and more. As highlighted in this review, patient derived organoid models are of specific interest here, as they pose a very defined experimental setup that can easily be adapted to explore ITH. Compared to mouse models (GEMMs and PDX), PDOs are time- and cost-effective, represent a purely human system, and are without the ethical implications of animal experiments while being more physiologically relevant than 2D cancer cell lines. PDO models are nevertheless no natural tumors which can exists solitary outside of an organism. The delicate interaction between tumor micro- and macroenviroment cannot be modeled in organoids without considerable effort.

While of course coming with the limitations of an in vitro system, PDOs are appropriate and convenient models not only to investigate ITH but also to apply high-throughput drug screenings to assess a tumor’s response to potential drug treatment, taking ITH into account. Just recently, a large consortium has shown the reliability of organoids as models and has published a comprehensive atlas of human organoid proteomics [94].

PDOs further have the potential for real personalized cancer medicine in a time- and cost-effective manner. Due to their ability to reflect the original tumor’s ITH and predict its drug response, organoids derived from an individual patient’s tumor can be used to screen for the drug treatment with the highest chance for positive treatment outcome, even without the need for additional, costly omics analyses. The results can be directly used for guiding clinical decision making to minimize the use of treatment regimen with high side-effects but potentially little positive impact. This approach to personalized cancer therapy is gaining increased attention and there are already efforts to make this a clinical reality, for example, through companies offering such personalized PDO models and drug screens as a service for patients and clinicians. For a wide clinical application, the establishment, maintenance, and screening of such models will need to become highly standardized. For increased clinical relevance, models will need to evolve further to recapitulate more complex cellular interactions with the stroma and the immune system.

Cancer treatment significantly improved over the last decades, mainly due to an enhanced understanding of the mechanisms leading to tumor formation and disease progression which were often accelerated by technological advancements. Current trends such as machine learning and artificial intelligence (AI) are on the horizon and show the need to facilitate the system to improve drug response prediction in patients by transferring information from cancer models [142]. If we hope to truly advance precision oncology, we need a pan-optic view on all facets of cancer biology, and we need to go beyond genomics. With the current technologies available, a personalized cancer drug response prediction is possible, paving the way to personalized oncology. “The Future Is Here—It Is Just Not Evenly Distributed” (William Gibson).

## Data Availability

Not applicable.
